# The Limited Role of Microbiological Culture and Sensitivity in the Management of Superficial Soft Tissue Abscesses

**DOI:** 10.1100/tsw.2006.215

**Published:** 2006-09-06

**Authors:** Muhammad N. Khan, Raghavan Vidya, Richard E. Lee

**Affiliations:** ^1^ Salisbury District Hospital, Salisbury, U.K.; ^2^ City Hospital, Birmingham, U.K.; ^3^ Queens Hospital, Burton on Trent, U.K.

**Keywords:** pus swabs, culture sensitivity, superficial soft tissue abscesses

## Abstract

The aim of this study was to assess the role of the routine practice of microbial culture and sensitivity at incision and drainage of superficial soft tissue abscesses. The case notes of 162 consecutive patients, selected from the microbiology database over a period of 1 year, were reviewed. All had incision and drainage of superficial soft tissue abscesses and included perianal, pilonidal, axillary, and breast abscesses. Patients with chronic wounds, recurrent abscesses, diabetes, pregnancy, and immunosuppression were excluded. The impact of pus culture and sensitivity (C/S) on management and clinical outcome was documented. Out of 162 patients, 97 were male (59.8%) and 65 were female (40.1%). Only 115 (70.9%) yielded positive cultures and 47 (29.1%) were sterile. The cultured microbial flora was predictable and sensitive to empirical antibiotics. In four patients, the results of microbial culture sensitivity showed microbial resistance to empirical antibiotics; however, it did not affect the management or the outcome for these patients. The routine practice of sending swabs for C/S after incision and drainage of superficial soft tissue abscesses does not contribute significantly towards patient management. Most patients are already on antibiotics prior to the referral and in the remainder, surgeons start antibiotics empirically. These broad-spectrum antibiotics cover the common pathogens involved, and there is no significant change in the antibiotic treatment after reviewing the culture reports following incision and drainage of uncomplicated superficial skin abscesses.

## INTRODUCTION

The standard treatment of an abscess is incision and drainage. The use of antibiotics depends on the clinical situation. It is a common practice to send pus swabs for culture and sensitivity after any surgical procedure where pus is drained. This may be helpful in growing an uncommon or resistant organism, and may help to target antibiotic therapy more precisely, but there are some procedures where the value is questionable[1,2,3]. Incision and drainage of superficial soft tissue abscesses may be one such example[4]. The aim of this study was to assess the role of microbial swabs following incision and drainage of superficial soft tissue abscesses and its use in antimicrobial therapy.

## PATIENTS AND METHODS

### Study Population

This study was carried out at a district general hospital. Between August 2002 and September 2003, the case notes of 162 consecutive patients, selected from the microbiology database, were reviewed. These patients had incision and drainage of superficial soft tissue abscesses.

### Methods

All patients had routine microbial swabs taken peroperatively. The data were obtained from the electronic patient records (HISS, hospital information support system) and the case notes. Age, sex, mode of admission (GP referral or emergency), previous course of antibiotics, results of culture swabs, and postoperative complications were recorded.

### Microbiology

All microbial swabs were taken using a single microbiological swab in a transport medium. Samples taken out of hours were refrigerated. The cultures were grown on blood agar and MacConkey agar for aerobic and anaerobic pathogens. Their sensitivities were determined using standard techniques.

### Postoperative Complications

The main complications recorded were readmission or reattendance to the hospital within 8 weeks, prolonged hospital stay (>48 h), wound infection with purulent discharge, and cellulitis. These patients were not routinely followed up, but the case notes were reviewed in detail for postoperative complications.

### Statistical Methods

Chi square test was used to analyse contingencies where appropriate. It was not possible to use complex statistical models because of the retrospective nature of the study. A $p$ value of $< 0.05$ was taken as significant.

## RESULTS

The study group was comprised of 97 (59.8%) men and 65 (40.1%) women with a median age of 38 years (range 24–73 years). In total, 63 perianal, 35 pilonidal, 29 breasts, 11 axillary, 6 groin abscesses, and 18 miscellaneous abscesses were drained. Most of the patients were discharged home within 24 h. Only six patients were required to stay for 48 h**.** Fifty-one patients were referred by their general practitioner and the rest presented in the emergency department.

A total of 162 swabs were cultured with a positive growth observed in 115 (70.9%). Positive culture rates in different types of abscesses are outlined in Table 1. There was no significant association between the positive culture rate and the type of abscess ($p = 1$). Of these, 47% (n = 55) yielded mainly aerobic species including *coliforms*, *gram-positive cocci* mainly *Staphylococcus aureus*, followed by *beta haemolytic streptococci* and *Streptococcus faecalis*. *S. milleri* was very rare and cultured from one specimen only. *MRSA* was not isolated. *Anaerobes* were mainly found in patients presenting with perianal and pilonidal abscesses (Table 1).

**TABLE I**
**Frequency of Different Microorganisms Grown from Each Group**

| Microorganisms | Perianal Abscesses | Pilonidal Abscesses | Breast Abscesses | Axillary Abscesses | Groin Abscesses | Others |
|---|---|---|---|---|---|---|
| Coliforms | 6 | 3 | 2 | 0 | 0 | 4 |
| Bacteriodes | 8 | 11 | 3 | 2 | 1 | 1 |
| *S. aureus* | 13 | 3 | 13 | 5 | 1 | 9 |
| Nonhaemolytic streptococci | | 1 | 1 | 0 | | 2 |
| Mixed growth | 17 | 10 | 2 | 0 | 2 | 3 |
| No growth | 19 | 7 | 8 | 4 | 2 | 5 |
| Total | 63 | 35 | 29 | 11 | 6 | 18 |
| Positive culture rate | 69.8% | 80.0% | 72.4% | 63.5% | 66.6% | 61.1% |

## Perianal Abscess

Most of the swabs from perianal abscesses grew *coliforms* and *bacteroides* (47.6%); 60% of patients had no antibiotic treatment, 35% had metronidazole, and 5% were treated with penicillin. All abscesses treated with primary drainage healed well except for two patients diagnosed with Crohn's disease. They presented with a recurrence and required examination under anaesthesia and further drainage.

## Pilonidal Abscess

Among the 35 patients presenting with pilonidal abscesses, 80% of cultures were positive and the commonest pathogens were *bacteroides* and *coliforms* (68%), with *S. milleri* and *Group A streptococci* grown in only one patient. Nine patients had postoperative antibiotics (metronidazole). There were four readmissions with recurrence. One of them had postoperative antibiotics. Prescribing postoperative antibiotics did not seem to have a statistically significant association with the recurrence ($p = 1$).

## Breast Abscess

*Staphylococcus aureus* and superficial skin flora were grown in the majority of the breast abscess swabs (51.7%), with *bacteroides* cultured in 20%. These were treated effectively with co-amoxiclav.

Table 2 explains the different antibiotics and their coverage of microorganisms along with the degree of resistance. *S. aureus* was sensitive to flucloxacillin. Only four isolates were penicillin resistant and were sensitive to ciprofloxacin. Three isolates were from perianal and one from a pilonidal abscess. The perianal abscess patients did not receive any antibiotic therapy after drainage. The pilonidal abscess

**TABLE 2**
**Antibiotics, Coverage of Microorganisms, and the Degree of Microbial Resistance**

| Antibiotics | Microorganisms Effectively Covered | No. of Resistant Organisms (Total No. of Organisms Cultured) |
|---|---|---|
| Flucloxacillin | *S. aureus* | *04 (59)* |
| Metronidazole | Bacteroides | *Nil (37)* |
| Cephalosporins | Coliforms | *02 (31)* |
| Ciprofloxacin | Coliforms | *Nil (31)* |
| Benzyl penicillin | β Haemolytic streptococci | *Nil (08)* |

patient was prescribed metronidazole for 5 days. There were no recorded complications or readmissions. No case of *MRSA* was seen. *Coliforms* were routinely sensitive to cephalosporins, with resistance seen in two cases that were sensitive to ciprofloxacin. Empirical choice of antibiotics for breast abscesses was co-amoxiclav, metronidazole for pilonidal abscesses, and metronidazole and cephalosporin for perianal abscesses, while for other superficial soft tissue abscesses benzyl penicillin and flucloxacillin were the choice. The results of C/S showed microbial resistance to empirical antibiotics in four cases. However, no change in the treatment was made and there were no adverse outcomes. Two of these cases did not have any postoperative antibiotics.

## DISCUSSION

Antibiotics have been effectively used to treat infections since the discovery of penicillin by Alexander Fleming in 1929[5]. While these so-called "magic bullets" have saved millions of lives, their widespread use has contributed to the emergence of resistant microorganisms[6,7]. Culture of the microorganisms and testing their sensitivity to different antibiotics has been the gold standard of treating infections, but in clinical practice, antibiotic therapy is often commenced empirically due to the time lag of 24–48 h in obtaining results of culture sensitivity[8].

The majority of patients with superficial soft tissue abscesses require incision and drainage only. The routine use of antibiotics in a superficial abscess has not proved beneficial[9,10,11]. Primary antibiotic therapy alone is ineffective in resolving the underlying infection, especially when pus is present. Antibiotics should only be used as an adjunct to surgical therapy for patients with comorbid conditions such as diabetes, valvular heart disease, or immunodeficiencies[12].

This study has demonstrated the current practice, in a district general hospital, of routine microbial swabs in patients undergoing incision and drainage of superficial soft tissue abscesses. A total of 162 swabs were taken, however only 70.9% of these yielded a positive culture. The microbial flora was common and had predictable sensitivities to antibiotics in the majority of the cases. Antibiotic treatment had generally not been altered by the culture sensitivity results with a major problem of delay in obtaining the reports as patients are usually discharged beforehand.

Depending on the anatomical site of the abscess, the cultured microorganisms were predictable and the results are similar to other published studies[13,14,15]. The culture growth was mainly comprised of mixed coliforms and bacteroides in perianal and pilonidal abscesses, whereas *Staphylococcus aureus* was the most common pathogen isolated from breast, head, and neck abscesses.

There is ongoing controversy regarding the relevance of microbiology in the management of perianal abscesses as several studies in the past have shown an association between the findings of gut-derived microorganisms and the subsequent development of a perianal fistula[16,17]. However, current standard practice is to reassess all patients after incision and drainage of perianal abscesses in the out-patients clinic after a period of 2–4 weeks, and if there is any question of a fistula, they undergo an examination under anaesthesia. Surgical assessment has been documented to be more sensitive and specific in detecting perianal fistulas as compared to the culture and sensitivity of the pus drained from these abscesses[18]. Nicholls et al. did not find the routine culture of pus from perianal abscesses helpful in the diagnosis of perianal fistula[19].

This study is not without limitations. In a retrospective study, it is very difficult to ascertain how effectively the culture results were followed up. The hospital records and case notes were reviewed to get this information, however in some cases, general practitioners and community nurses may have followed up these results and prescribed antibiotic therapy as an outpatient. In this study, we used hospital stay and readmission as a marker of postoperative complications. However, in the management of superficial abscesses, if the patient has persistent trouble, he is likely to present to the general practitioner who may prescribe further antibiotics or carry out a drainage procedure. Similarly, antibiotic therapy prior to the drainage of an abscess may alter the culture results. The inclusion of out patients' data from the general practitioner of these patients would have strengthened the results.

The standard treatment for an abscess remains incision and drainage. The use of postoperative antibiotics is guided by the clinical picture and the presence of comorbid conditions. The microbial flora is predictable and sensitive to empirical antibiotics. In the present economic climate with limited resources, the routine practice of culture and sensitivity also does not appear to be cost effective. Culturing a microbial swab costs between £25–30 and each requested antibiotic sensitivity would cost an additional £5 per set per organism[20]. This is in addition to the manpower resource used in the procedure. A total of 162 swabs were processed in our unit in a year, at a marginal cost in the region of £5000. This study suggests a limited role for the routine practice of obtaining microbial swabs at incision and drainage of superficial soft tissue abscesses. A modified approach should be adopted for selected cases particularly in patients who are immunosuppressed and to monitor the hospital infection control surveillance.

## ACKNOWLEDGEMENTS

We are immensely grateful to Mr. B.J. Moran, Mr. T.D. Cecil, and Miss M. Farquharson (Consultant Surgeons Basingstoke) for their help in reviewing the manuscript and useful suggestions. We would also like to thank Dr. James Paton (microbiologist Queens Hospital Burton) for his help in accessing the patient records and culture results.

## References

1. Bowlerm P., Duerdenm B., and Armstrong, D. (2001) Wound microbiology and associated approaches to wound management. *Clin. Microbiol. Rev.* **14(2),** 244–269.
2. Lawrence, C. (1985) The bacteriology of burns. *J. Hosp. Acquir. Infect*. **6**, 3–17 (Suppl).
3. Cooper, R. and Lawrence, J.C. (1996) The isolation and identification of bacteria from wounds. *J. Wound Care* **5(7),** 335–340.
4. Garcea, G., Lloyd, T., Jacobs, M., Cope, A., Swann, A., and Berry, D. (2003) Role of microbiological investigations in the management of non-perineal cutaneous abscesses. *Postgrad. Med. J.* **79**, 519–521.
5. Wainwright, M. (1990) *Miracle Cure: The Story of Penicillin and the Golden Age of Antibiotics.* Basil Blackwell, Oxford.
6. Pallasch, T.J. (2003) Antibiotic resistance. *Dent. Clin. North Am.* **47(4),** 623–639.
7. Anderson, D.I. (2003) Persistence of antibiotic resistant bacteria. *Curr. Opin. Microbiol.* **6(5),** 452–456.
8. Garcia-de Lomas, J. and Navarro, D. (1997) New directions in diagnostics. Pediatr. Infect. Dis. J. **16(3 Suppl),** S43–48.
9. Macfie, J. and Harvey, J. (1977) The treatment of acute superficial abscesses: a prospective clinical trial. *Br. J. Surg.* **64(4),** 264–266.
10. Meislin, H.W., Lerner, S.A., Graves, M.H., Mcghee, M.D., Kocka, F.E., et al. (1977) Cutaneous abscesses. Anaerobic and aerobic bacteriology and out patient management. *Ann. Intern. Med.* **87(2),** 145–149.

11. Stewart, M.P., Laing, M.R., and Krukowski, Z.H. (1985) Treatment of acute abscesses by incision, curettage and primary suture without antibiotics: a controlled trial. *Br. J. Surg.* **72(1),** 66–67.
12. Dougherty, S.H. (1997) Antimicrobial culture and susceptibility testing has little value for routine management of secondary bacterial peritonitis. *Clin. Infect. Dis.* **25(Suppl 2),** S258–261.
13. Llera, J.L., Levy, R.C., and Staneck, J.L. (1984) Cutaneous abscesses: natural history and management in an out patient facility. *J. Emerg. Med.* **1(6),** 489–493.
14. Halvorson, G.D., Halvorson, J.E., and Iserson, K.V. (1985) Abscess incision and drainage in the emergency department. I. *J. Emerg. Med.* **3(3),** 227–232.
15. Brook, I. and Frazier, E.H. (1997) The aerobic and anaerobic bacteriology of peri rectal abscesses. *J. Clin. Microbiol.* **35,** 2974–2976.
16. Grace, R.H., Harper, I.A., and Thompson, R.G. (1982) Anorectal sepsis: microbiology in relation to fistula in ano. *Br. J. Surg.* **69**, 401–403.
17. Kufahl, J.W. and Andreasen, J.J. (1992) Microbiology related to anal abscess complicated with fistula formation. *Ugeskr. Laeger*. **154,** 1428–1429.
18. Lunniss, P.J. and Phillips, R.K. (1994) Surgical assessment of acute anorectal sepsis is a better predictor of fistula than microbiological analysis. *Br. J. Surg.* **81(3),** 368–369.
19. Nicholls, G., Heaton, N.D., and Lewis, A.M. (1990) Use of bacteriology in anorectal sepsis as an indicator of anal fistula: an experience in a district general hospital. *J. R. Soc. Med.* **83(10),** 625–626.
20. Gilchrist, B. (2000) Taking a wound swab. *Nurs. Times* **96(4 Suppl),** 2.
